# Supplementary material for: Combining Fe nanoparticles and pyrrole-type Fe-N4 sites on less-oxygenated carbon supports for electrochemical CO2 reduction
Source: Nat Commun. 2023 Aug 22;14:5108. doi: 10.1038/s41467-023-40667-2 (PMC10444801; doi:10.1038/s41467-023-40667-2)
Supplement: Supplementary file 1 — Supplementary Information [file 41467_2023_40667_MOESM1_ESM.pdf]

Supplementary Information

Combining Fe nanoparticles and pyrrole-type Fe-N_4 sites on less-oxygenated carbon supports for electrochemical CO_2 reduction

Cai Wang,[1] Xiaoyu Wang,[1] Houan Ren,[1] Yilin Zhang,[1] Xiaomei Zhou,[1] Jing Wang,[1] Qingxin Guan,[1] Yuping Liu,[1] Wei Li*,[1]

[1]State Key Laboratory of Elemento-Organic Chemistry, Key Laboratory of Advanced Energy Materials Chemistry (Ministry of Education), College of Chemistry, Nankai University, Tianjin 300071, China

*E-mail: weili@nankai.edu.cn

Supplementary Figures and Tables

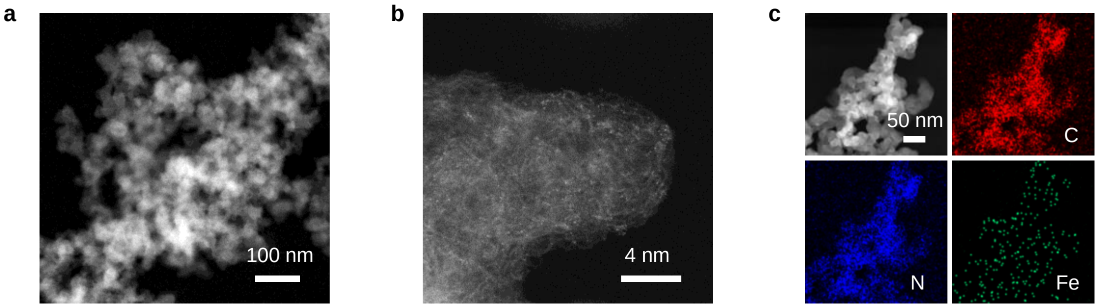

Figure S1. (a) HAADF-TEM, (b) aberration-corrected HAADF-STEM, and (c) EDS mapping of Fe-pdN-C(O).

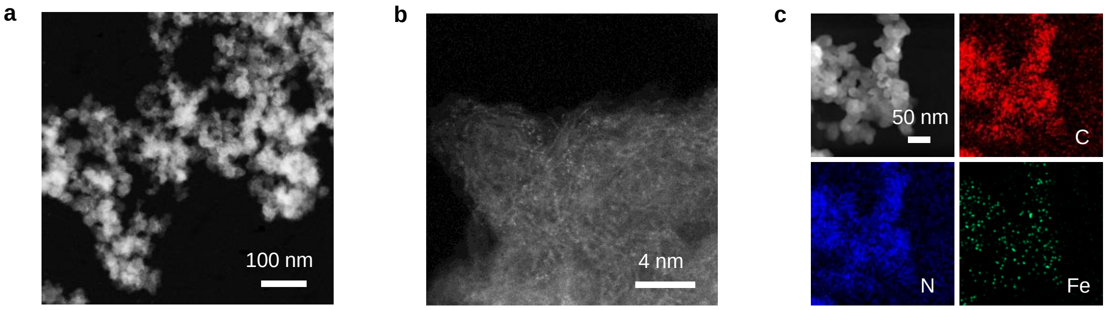

Figure S2. (a) HAADF-TEM, (b) aberration-corrected HAADF-STEM, and (c) EDS mapping of Fe-poN-C(O).

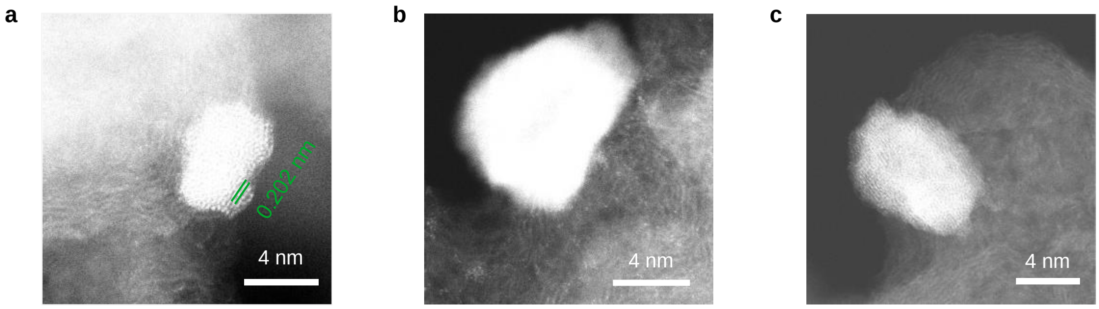

Figure S3. Aberration-corrected HAADF-STEM of Fe-poN-C/Fe at other positions.

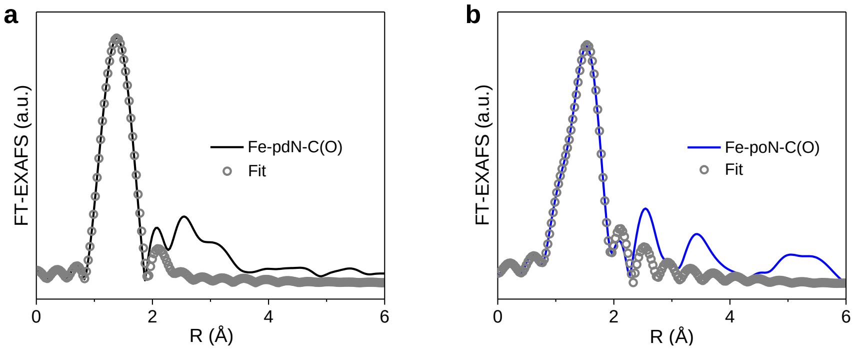

Figure S4. The fitting curve of FT-EXAFS for Fe-pdN-C(O) and Fe-poN-C(O).

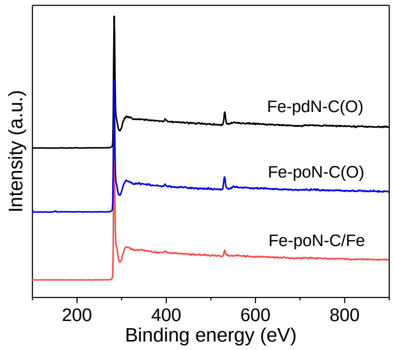

Figure S5. Full XPS spectra of Fe-pdN-C(O), Fe-poN-C(O) and Fe-poN-C/Fe.

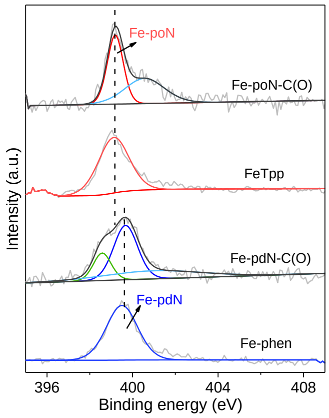

Figure S6. N 1s XPS spectra of Fe-pdN-C(O), Fe-poN-C(O), Fe-phen complex and FeTpp.

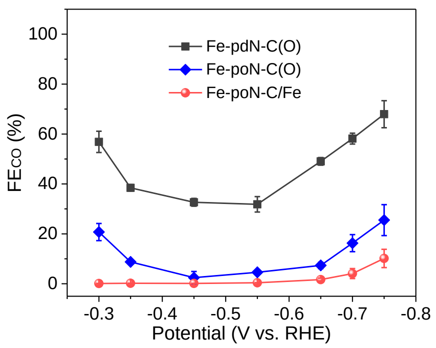

Figure S7. Faraday efficiency of H_2 for Fe-pdN-C(O), Fe-poN-C(O) and Fe-poN-C/Fe. Error bars represent the standard deviation of three independent measurements.

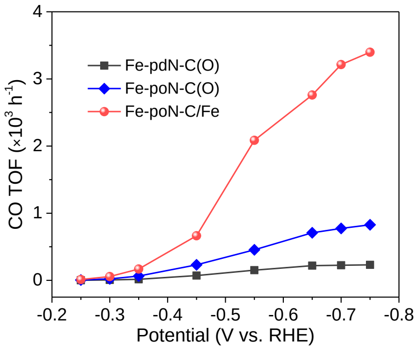

Figure S8. The calculated CO TOF of Fe-pdN-C(O), Fe-poN-C(O) and Fe-poN-C/Fe.

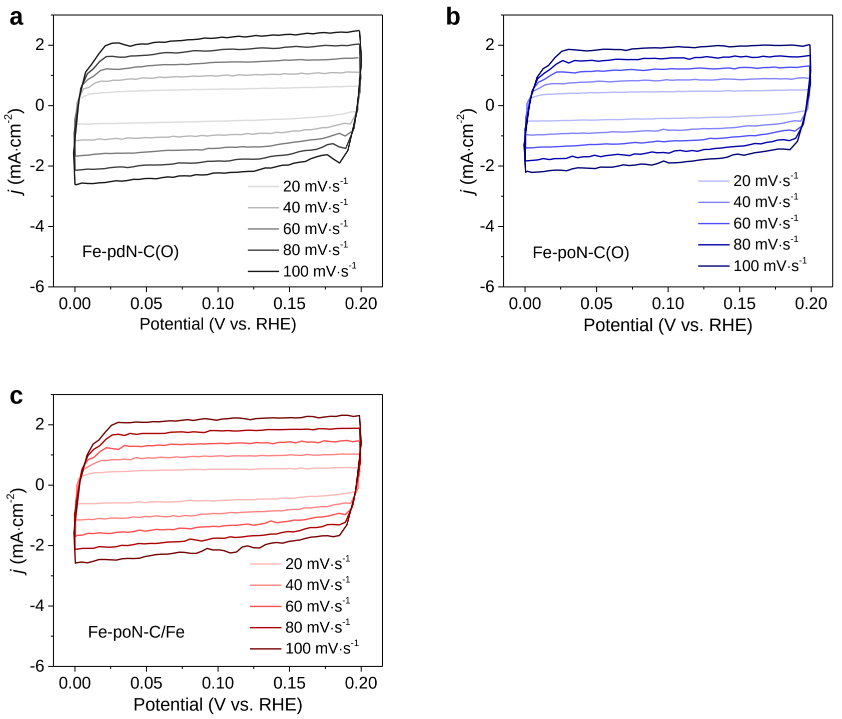

Figure S9. Cyclic voltammograms with different scan rate of (a) Fe-pdN-C(O), (b) Fe-poN-C(O) and (c) Fe-poN-C/Fe.

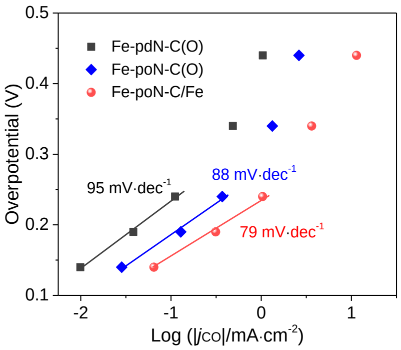

Figure S10. The Tafel plots of Fe-pdN-C(O), Fe-poN-C(O) and Fe-poN-C/Fe.

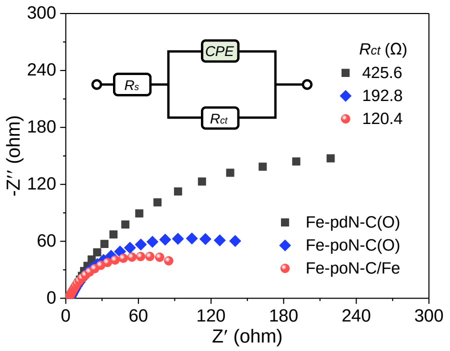

Figure S11. The EIS spectra of Fe-pdN-C(O), Fe-poN-C(O) and Fe-poN-C/Fe. Inset is the equivalent series circuit.

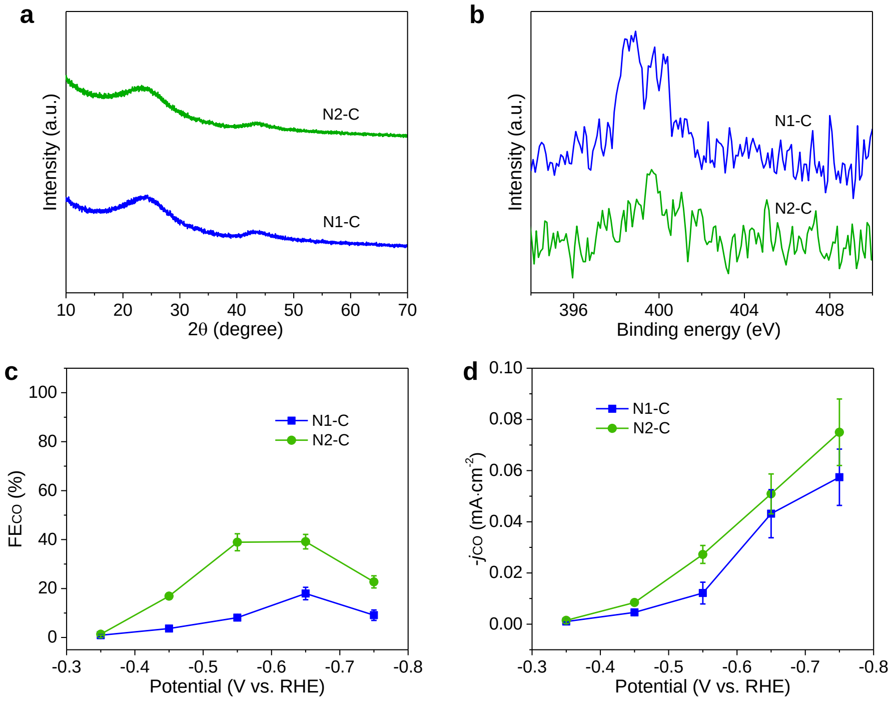

Figure S12. (a) XRD patterns, (b) N 1s spectra, (c) FE_{CO} and (d) j_{CO} of N1-C and N2-C. Error bars in (c) and (d) represent the standard deviation of three independent measurements.

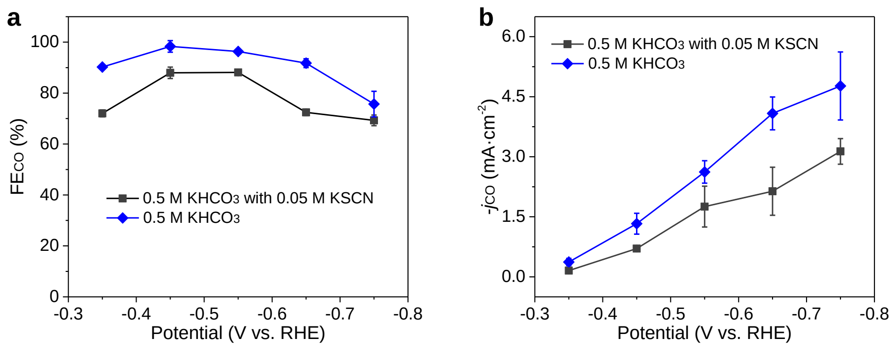

Figure S13. (a) FE_{CO} and (b) j_{CO} of Fe-poN-C(O) with and without 0.05 M KSCN. Error bars in represent the standard deviation of three independent measurements.

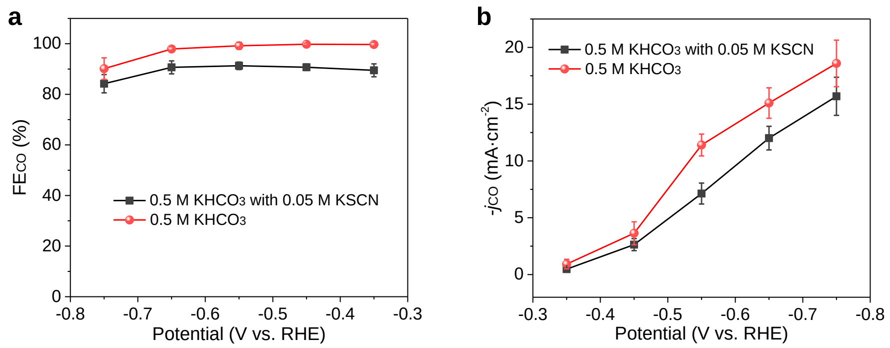

Figure S14. (a) FE_{CO} and (b) j_{CO} of Fe-poN-C/Fe with and without 0.05 M KSCN. Error bars in represent the standard deviation of three independent measurements.

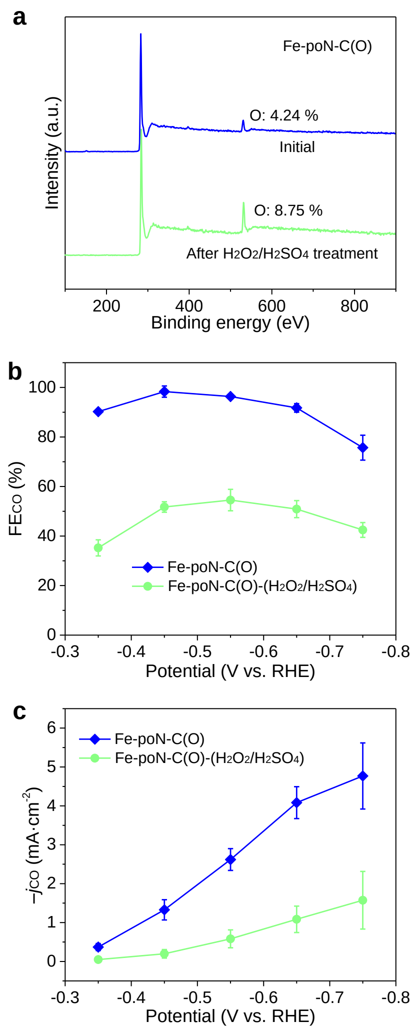

Figure S15. (a) Full XPS spectra, (b) FE_{CO} and (c) j_{CO} of Fe-pdN-C(O) before and after H_2O_2/H_2SO_4 treatment. Error bars in (b) and (c) represent the standard deviation of three independent measurements.

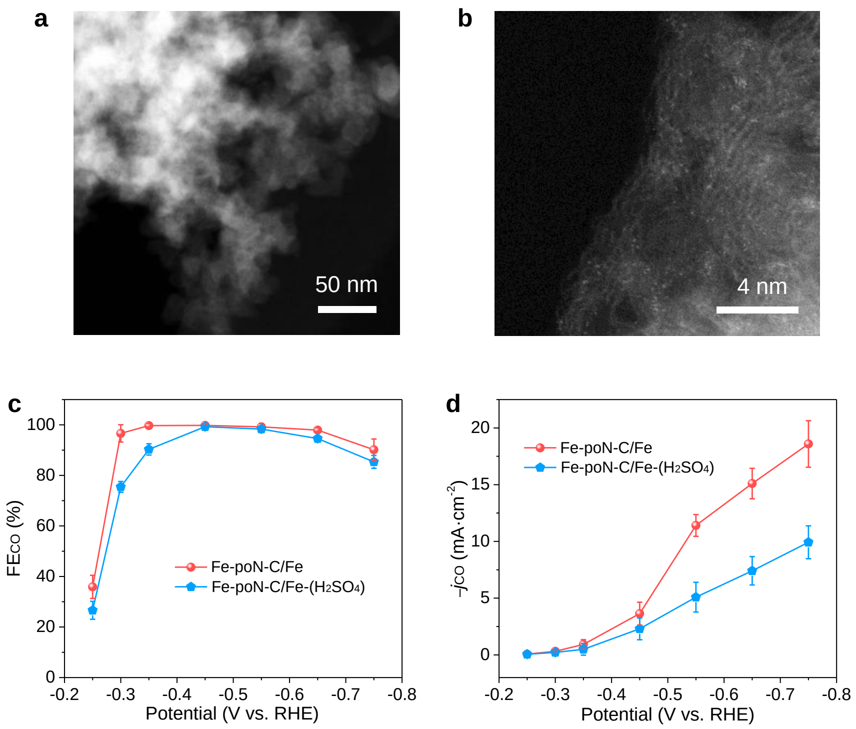

Figure S16. (a) HAADF-TEM and (b) aberration-corrected HAADF-STEM of Fe-poN-C/Fe after H_2SO_4 treatment. (c) FE_{CO} and (d) j_{CO} of Fe-poN-C/Fe before and after H_2SO_4 treatment. Error bars in (c) and (d) represent the standard deviation of three independent measurements.

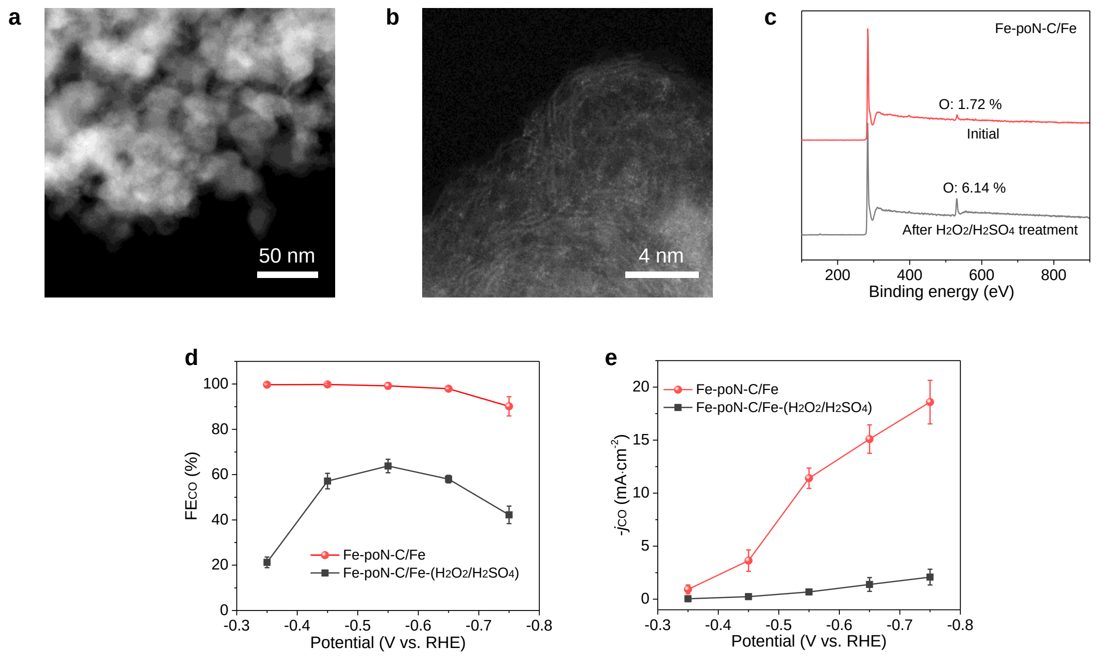

Figure S17. (a) HAADF-TEM, (b) aberration-corrected HAADF-STEM, and (c) full XPS spectra of Fe-poN-C/Fe after H_2O_2/H_2SO_4 treatment. (d) FE_{CO} and (e) j_{CO} of Fe-poN-C/Fe before and after H_2O_2/H_2SO_4 treatment. Error bars in (d) and (e) represent the standard deviation of three independent measurements.

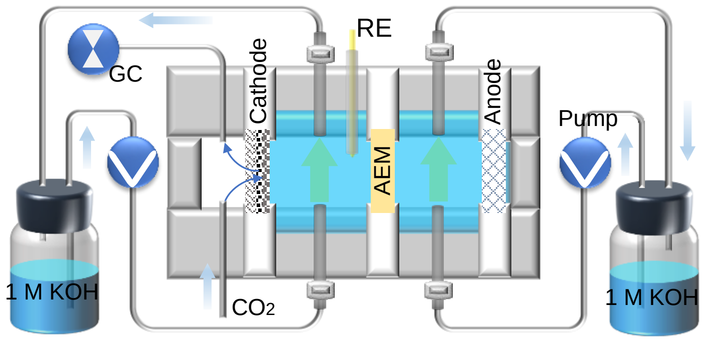

Figure S18. Schematic of a gas-fed flow cell configuration.

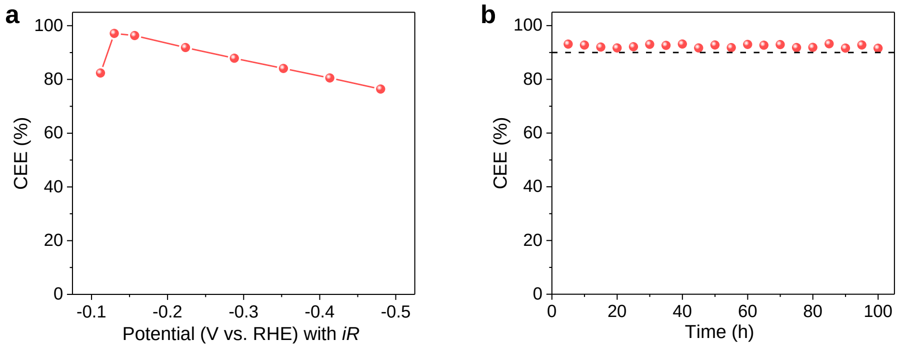

Figure S19. (a) The calculated CEE of Fe-poN-C/Fe for CO production under different potential with *iR* correction. (b) The calculated CEE of Fe-poN-C/Fe for CO production under stability tests.

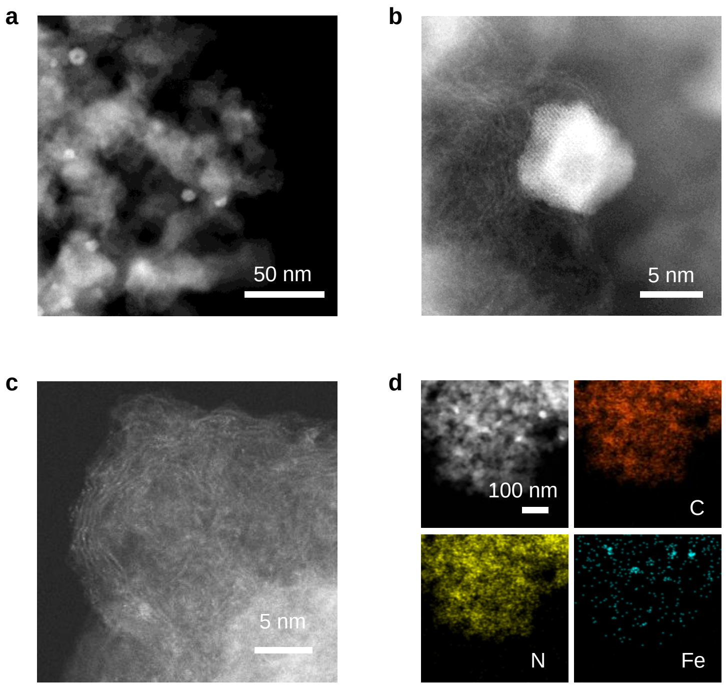

Figure S20. (a) HAADF-TEM, (b,c) aberration-corrected HAADF-STEM, and (d) EDS mapping of Fe-poN-C/Fe after long-term electrolysis.

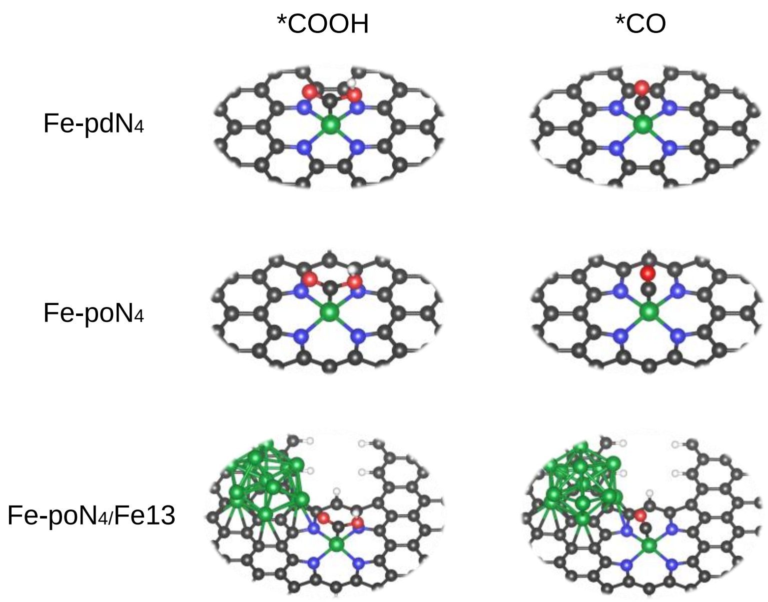

Figure S21. Simulated probable pathways of *COOH to *CO on the optimized structure of Fe-pdN_4, Fe-poN_4 and Fe-poN_4/Fe13.

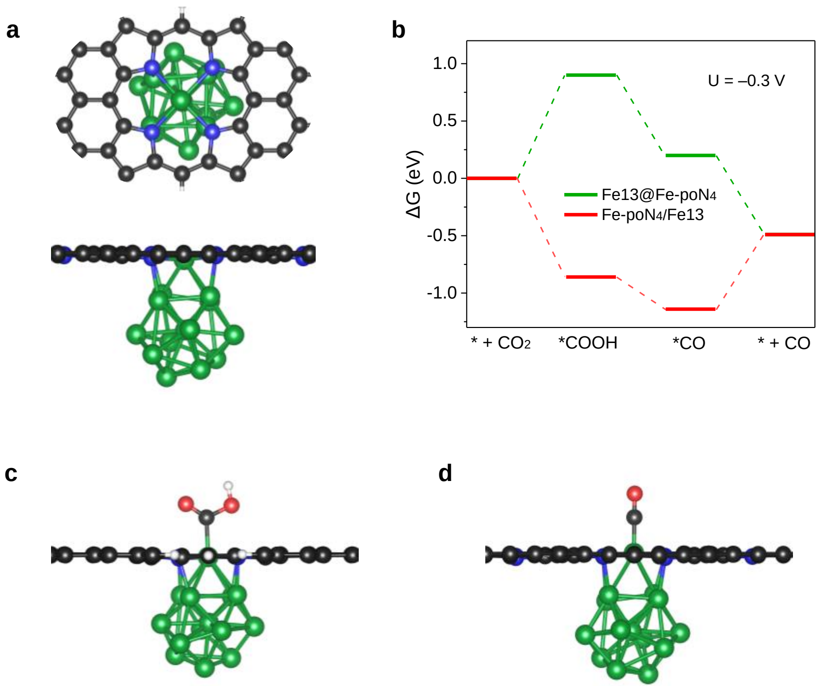

Figure S22. (a) The optimized structures of Fe13@Fe-poN_4. (b) Free energy diagrams of ECR at −0.3 V. Simulated probable pathways of *COOH (c) to *CO (d) on the optimized structure of Fe13@Fe-poN_4.

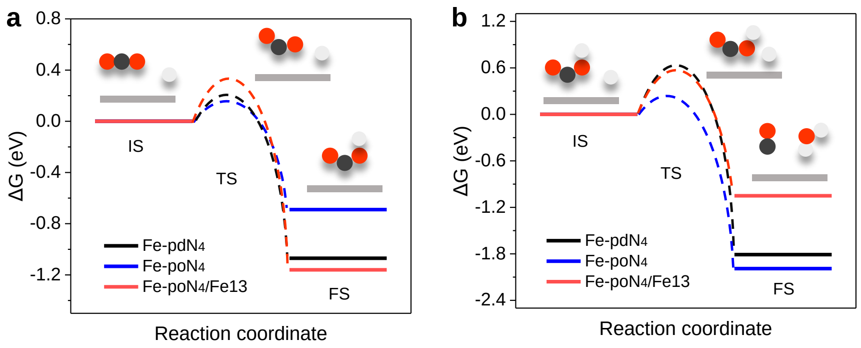

Figure S23. The activation energies for Fe-pdN_4, Fe-poN_4 and Fe-poN_4/Fe13 along the CO_2 reduction pathway. Insets are simple structures of initial state (IS), transition state (TS) and final state (FS).

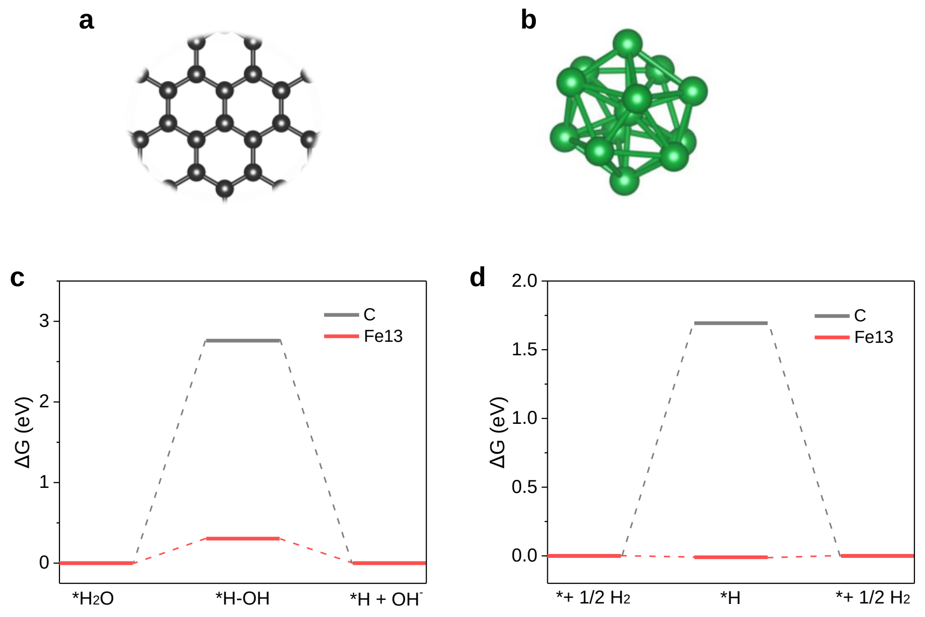

Figure S24. The models of (a) C and (b) Fe13. Free energy diagram for (c) dissociative water reaction and (d) HER on the models of C and Fe13.

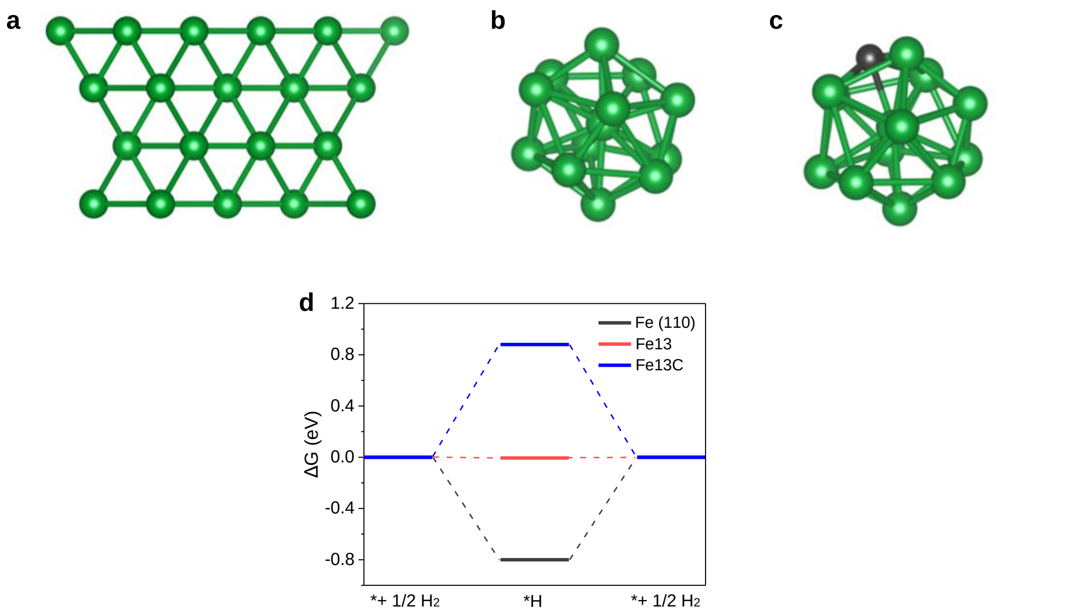

Figure S25. The model of (a) Fe (110), (b) Fe13 and (c) Fe13C. (d) Free energy diagrams of HER on the models of Fe (110), Fe13 and Fe13C.

Fe (110) represents Fe NPs with large size, Fe13 clusters represents Fe NPs with small size, Fe13C represents Fe NPs after the incorporation of carbon. Obviously, the size of Fe NPs has a significant impact on the free energy change for *H formation, and the HER on Fe NPs became difficult after the incorporation of carbon.

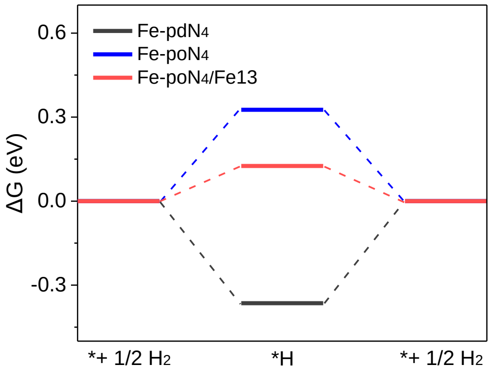

Figure S26. Free energy diagrams of HER on the models of Fe-pdN_4, Fe-poN_4 and Fe-poN_4/Fe13.

Table S1. EXAFS data fitting results of prepared catalysts.

Samples	Shell	N [a]	R (Å) [b]	σ^2 (Å^2·10^{-3}) [c]	ΔE_0 (eV) [d]	*R* factor (%)
Fe-pdN-C(O)	Fe-N	4.1	1.99	9.8	-2.7	0.9
Fe-poN-C(O)	Fe-N	4.2	2.11	11.1	-3.3	0.5
Fe-poN-C/Fe	Fe-N	3.9	1.91	9.0	-5.2	1.2
	Fe-Fe	1.3	2.59	9.9	4.8	

[a] *N*: coordination numbers; [b] *R*: bond distance; [c] σ^2: Debye-Waller factors; [d] ΔE_0: the inner potential correction. *R* factor: goodness of fit. S_0^2 is the amplitude reduction factor, set as 0.84/0.90 for Fe-N/Fe-Fe.

Table S2. XPS analysis results of prepared catalysts.

Catalysts	C (at. %)	N (at. %)	O (at. %)	Fe (at. %)
Fe-pdN-C(O)	93.79	1.56	4.24	0.41
Fe-poN-C(O)	94.24	1.32	4.08	0.36
Fe-poN-C/Fe	96.73	1.20	1.72	0.35

Table S3. Data and references from Figure 3c for previous ECR to CO reports on ECR performance in an H-type cell.

No. in Graph Figure 3c	Catalyst	Electrolyte	Overpotential (V)	FE_{CO} (%)	References
**--**	**Fe-poN-C/Fe**	**0.5 M $KHCO_3$**	**0.24**	**99.7**	**This work**
1	FeN_5	0.1 M $KHCO_3$	0.35	97	1
2	Fe_1-NSC	0.5 M $KHCO_3$	0.37	98.6	2
3	Fe_1NC/S_1-1000	0.5 M $KHCO_3$	0.39	96	3
4	Fe-N-C	0.5 M $KHCO_3$	0.39	93.5	4
5	Fe-CNPs	1 M $KHCO_3$	0.47	98.8	5
6	H_2-FeN_4/C	0.1 M $NaHCO_3$	0.49	97	6
7	Fe-N/O-C(MZ)	0.1 M $KHCO_3$	0.47	96	7
8	Fe_2NPC	0.1 M $KHCO_3$	0.49	96	8
9	Fe-N-C	0.1 M $KHCO_3$	0.47	93	9
10	FeN_4/C	0.1 M $KHCO_3$	0.49	93	10
11	$Fe_{0.5}d$	0.5 M $NaHCO_3$	0.39	80	11
12	Fe/NG-750	0.1 M $KHCO_3$	0.49	80	12
13	Oxide-derived Au	0.5 M $NaHCO_3$	0.24	96	13
14	C-Au-500 NWs	0.5 M $KHCO_3$	0.24	94	14
15	Ag nanocorals	0.1 M $KHCO_3$	0.37	95	15
16	np-Ag	0.5 M $KHCO_3$	0.39	90	16
17	Pd NPs	0.1 M $KHCO_3$	0.58	90	17

18	Tri-Ag-NPs	0.1 M $KHCO_3$	0.746	96.8	18
19	Mn-C_3N_4/CNT	0.5 M $KHCO_3$	0.44	98.8	19
20	CoPc/CNT	0.1 M $KHCO_3$	0.48	92	20
21	Cu-S_1N_3/Cu_x	0.1 M $KHCO_3$	0.54	99.7	21
22	NiSA/NP	0.5 M $KHCO_3$	0.69	99	22

Table S4. Data and references from Figure 4c for previous ECR to CO reports on ECR performance in a flow cell.

No. in Graph Figure 4c	Catalyst	Electrolyte	Overpotential (mV)	FE_{CO} (%)	References
**--**	**Fe-poN-C/Fe**	**1 M KOH**	**41 (without iR)**<br>**21 (with iR)**	**99.2**	**This work**
1	NiN_x/NCNT	1 M KOH	166	99.5	23
2	NiSA/NP	1 M KOH	341	99.6	22
3	NiPc-MDE	1 M $KHCO_3$	500	99.8	24
4	MWNT/PyPBI/Au	2 M KOH	167	98.3	25
5	Sn/NCNFs	1 M KOH	191	95.6	26
6	CoPc2	1 M KOH	411	97	27
7	$Fe_1N_2O_2$/NC	1 M KOH	91	91	28
8	C/Ag/PTFE	1 M KOH	301	90	29
9	Ni-N_4/C-NH_2	1 M KOH	391	84.5	30
10	NiSA/PCFM	0.5 M $KHCO_3$	491	80	31

Table S5. Data and references from Figure 4d for previous ECR to CO reports on CEE value.

No. in Graph Figure 4d	Catalyst	Electrolyte	Overpotential (mV)	CEE (%)	References
**--**	**Fe-poN-C/Fe**	**1 M KOH**	**21**	**97.1**	**This work**
1	NiN_x/NCNT	1 M KOH	166	86.9	23
2	Fe_1-NSC	1 M KOH	240	83.8	2
3	100 nm Ag	7 M KOH	300	81.5	32
4	$Fe_1N_2O_2$/NC	1 M KOH	390	77.2	28
5	$Sn_{0.3}Ti_{0.7}O_2$	0.5 M $KHCO_3$	430	71.5	33
6	CoPc2	1 M KOH	200	80.9	27
7	Ag/MWCNT	1 M KOH	160	75	34
8	MWNT/PyPBI/Au	2 M KOH	160	74.3	25

Supplementary References

1. Zhang, H. et al. Graphene - supported single - atom FeN_5 catalytic site for efficient electrochemical CO_2 reduction. *Angew. Chem. Int. Ed.* **2019***, 131,* 15013-15018.

2. Chen, S. et al. Unveiling the proton-feeding effect in sulfur-doped Fe-N-C single-atom catalyst for enhanced CO_2 electroreduction. *Angew. Chem. Int. Ed.* **2022**, *61*, e202206233.

3. Wang, T. et al. Gas diffusion strategy for inserting atomic iron sites into graphitized carbon supports for unusually high-efficient CO_2 electroreduction and high-performance Zn-CO_2 batteries. *Adv. Mater.* **2020**, *32*, 2002430.

4. Qin, X., Zhu, S., Xiao, F., Zhang, L., Shao, M. Active sites on heterogeneous single-iron-atom electrocatalysts in CO_2 reduction reaction. *ACS Energy Lett.* **2019***, 4,* 1778-1783.

5. Hu, C. et al. Porosity-induced high selectivity for CO_2 electroreduction to CO on Fe-doped ZIF-derived carbon catalysts. *ACS Catal.* **2019***, 9,* 11579-11588.

6. Liu, C. et al. Constructing FeN_4/graphitic nitrogen atomic interface for high-efficiency electrochemical CO_2 reduction over a broad potential window. *Chem* **2021**, *7*, 1-11.

7. Wang, X. et al. Hierarchically micro- and meso-porous Fe-N_4O-doped carbon as robust electrocatalyst for CO_2 reduction. *Appl. Catal. B-Environ.* **2020**, *266*, 118630.

8. Zhao, X. et al. Highly efficient electrochemical CO_2 reduction on a precise homonuclear diatomic Fe-Fe catalyst. *ACS Catal.* **2022**, *12*, 11412-11420.

9. Pan, F. et al. Unveiling active sites of CO_2 reduction on nitrogen-coordinated and atomically dispersed iron and cobalt catalysts. *ACS Catal.* **2018**, *8*, 3116-3122.

10. Li, X. et al. Isolated FeN_4 sites for efficient electrocatalytic CO_2 reduction. *Adv. Sci.* **2020**, 2001545.

11. Huan, T. N. et al. Electrochemical reduction of CO_2 catalyzed by Fe-N-C materials: a structure-selectivity study. *ACS Catal.* **2017**, *7*, 1520-1525.

12. Zhang, C. et al. Electrochemical CO_2 reduction with atomic iron-ispersed on nitrogen-doped graphene. *Adv. Energy Mater.* **2018**, *8*, 1703487.

13. Chen, Y., Li, C. W., Kanan, M. W. Aqueous CO_2 reduction at very low overpotential on oxide-derived Au nanoparticles. *J. Am. Chem. Soc.* **2012**, *134*, 19969-19972.

14. Zhu, W. et al. Active and selective conversion of CO_2 to CO on ultrathin Au nanowires. *J.*

Am. Chem. Soc. **2014**, *136*, 16132-16135.

15. Hsieh, Y. C., Senanayake, S. D., Zhang, Y., Xu, W., Polyansky, D. E. Effect of chloride anions on the synthesis and enhanced catalytic activity of silver nanocoral electrodes for CO_2 electroreduction. *ACS Catal.* **2015**, *5*, 5349-5356.

16. Lu, Q. et al. Selective and efficient electrocatalyst for carbon dioxide reduction. *Nat. Commun.* **2014**, *5*, 3242.

17. Gao, D. et al. Size-dependent electrocatalytic reduction of CO_2 over Pd nanoparticles. *J. Am. Chem. Soc.* **2015**, *137*, 4288-4291.

18. Liu, S. B. et al. Shape-dependent electrocatalytic reduction of CO_2 to CO on triangular silver nanoplates. *J. Am. Chem. Soc.* **2017***, 139,* 2160-2163.

19. Feng, J. et al. A Mn-N_3 single-atom catalyst embedded in graphitic carbon nitride for efficient CO_2 electroreduction. *Nat. Commun.* **2020**, *1*, 4341.

20. Zhang, X. et al. Highly selective and active CO_2 reduction electrocatalysts based on cobalt phthalocyanine/carbon nanotube hybrid structures. *Nat. Commun.* **2017**, *8*, 14675.

21. Li, F. et al. A tandem strategy for enhancing electrochemical CO_2 reduction activity of single-atom Cu-S_1N_3 catalysts via integration with Cu nanoclusters. *Angew. Chem. Int. Ed.* **2021**, *60*, 24022-24027.

22. Ren, W. et al. Electronic regulation of nickel single atoms by confined nickel nanoparticles for energy-efficient CO_2 electroreduction. *Angew. Chem. Int. Ed.* **2022**, *61*, e202203335.

23. Zhao, R. et al. Partially nitrided Ni nanoclusters achieve energy-efficient electrocatalytic CO_2 reduction to CO at ultralow overpotential. *Adv. Mater.* **2022***,* 2205262.

24. Zhang, X. et al. Molecular engineering of dispersed nickel phthalocyanines on carbon nanotubes for selective CO_2 reduction. *Nat. Energy* **2020**, *5*, 684-692.

25. Verma, S. et al. Insights into the low overpotential electroreduction of CO_2 to CO on a supported gold catalyst in an alkaline flow electrolyzer. *ACS Energy Letters* **2017***, 3,* 193-198.

26. Hu, X. et al. Boosting industrial-level CO_2 electroreduction of n-doped carbon nanofibers with confined tin-nitrogen active sites via accelerating proton transport kinetics. *Adv. Funct. Mater.* **2022***,* 2208781.

27. Wang, M. et al. CO_2 Electrochemical catalytic reduction with a highly active cobalt phthalocyanine. *Nat. Commun.* **2019**, *10*, 3602.

28. Zhao, D. et al. Atomic-level engineering $Fe_1N_2O_2$ interfacial structure derived from oxygen-abundant metal-organic frameworks to promote electrochemical CO_2 reduction. *Energy Environ. Sci.* **2022**, *15*, 3795-3804.

29. Dinh, C. T., Arquer, F. P. G. D., Sinton, D., Sargent, E. H. High Rate, Selective and stable electroreduction of CO_2 to CO in basic and neutral media. *ACS Energy Lett.* **2018**, *3*, 2835-2840.

30. Chen, Z. et al. Amination strategy to boost the CO_2 electroreduction current density of M-N/C single-atom catalysts to the industrial application level. *Energy Environ. Sci.* **2021**,*14*, 2349-2356.

31. Yang, H. et al. Carbon dioxide electroreduction on single-atom nickel decorated carbon membranes with industry compatible current densities. *Nat. Commun.* **2020**, *11*, 593.

32. Gabardo, C. M. et al. Combined high alkalinity and pressurization enable efficient CO_2 electroreduction to CO. *Energy Environ. Sci.,* **2018**,*11,* 2531-2539.

33. Wen, G. et al. Ternary Sn-Ti-O electrocatalyst boosts the stability and energy efficiency of CO_2 reduction. *Angew. Chem. Int. Ed.* **2020***, 59,* 12860-12867.

34. Ma, S. et al. Carbon nanotube containing Ag catalyst layers for efficient and selective reduction of carbon dioxide. *J. Mater. Chem. A,* **2016***, 4,* 8573-8578.
